# Gynecologic Malignancies in Children and Adolescents: How Common is the Uncommon?

**DOI:** 10.3390/jcm10040722

**Published:** 2021-02-12

**Authors:** Christoph Wohlmuth, Iris Wohlmuth-Wieser

**Affiliations:** 1 Department of Obstetrics and Gynecology, Paracelsus Medical University Salzburg, 5020 Salzburg, Austria; 2 Department of Dermatology, Paracelsus Medical University Salzburg, 5020 Salzburg, Austria; iris.wohlmuth-wieser@outlook.com

**Keywords:** gynecologic malignancies, pediatric cancer, gynecologic cancer, pediatric, adolescent, female

## Abstract

The aim of this study is to assess the projected incidence and prognostic indicators of gynecologic malignancies in the pediatric population. In this population-based retrospective cohort study, girls ≤18 years with ovarian, uterine, cervical, vaginal and vulvar malignancies diagnosed between 2000 and 2016 were identified from the Surveillance, Epidemiology and End Results (SEER)-18 registry. The Kaplan–Meier method was used to analyze overall survival (OS). The age-adjusted annual incidence of gynecologic malignancies was 6.7 per 1,000,000 females, with neoplasms of the ovary accounting for 87.5%, vagina 4.5%, cervix 3.9%, uterus 2.5% and vulva 1.6% of all gynecologic malignancies. Malignant germ-cell tumors represented the most common ovarian neoplasm, with an increased incidence in children from 5–18 years. Although certain subtypes were associated with advanced disease stages, the 10-year OS rate was 96.0%. Sarcomas accounted for the majority of vaginal, cervical, uterine and vulvar malignancies. The majority of vaginal neoplasms were observed in girls between 0–4 years, and the 10-year OS rate was 86.1%. Overall, gynecologic malignancies accounted for 4.2% of all malignancies in girls aged 0–18 years and the histologic subtypes and prognosis differed significantly from patients in older age groups.

## 1. Introduction

Malignant neoplasms are the fifth most common cause of death among children and adolescents aged 0–18 years and the third most common cause when excluding infants <1 year [1,2]. Gynecologic malignancies, including ovarian, uterine, cervical, vaginal and vulvar cancer, have been well described in the general population, and treatment guidelines have been tailored to clinical-, stage- and histology-based characteristics [3,4,5,6]. With successful advances in cancer therapy, survival is improving and fertility-preserving treatment options have become a critical aspect in the management of women with cancer in reproductive ages [7]. This aspect is also critical in young girls with cancer, but data on gynecologic malignancies in childhood remains scarce. While prospective trials including GOG-10, 45, 90 and 116 allowed for the inclusion of children and adolescents with certain gynecologic cancer subtypes and analyses from pediatric oncology cooperative groups assessed treatment outcome, there is a paucity of epidemiologic data and real-world prognosis [8,9,10,11,12,13,14,15].

The aim of this study was to systematically describe the epidemiology, histologic types and prognosis of gynecologic malignancies in this age group.

## 2. Materials and Methods

### 2.1. Study Population

The Surveillance, Epidemiology and End Results (SEER) database of the National Cancer Institute was used to identify cases of gynecologic malignancies in children and adolescents. The SEER-18 population (including Atlanta, Connecticut, Detroit, Hawaii, Iowa, New Mexico, San Francisco-Oakland, Seattle-Puget Sound, Utah, Los Angeles, San Jose-Monterey, Rural Georgia, the Alaska Native Tumor Registry, Greater California, Greater Georgia, Kentucky, Louisiana and New Jersey) from its November 2018 submission was used. This was limited to cases from 2000–2016 [16].

Patients aged 0–18 years with a diagnosis of invasive cancer of the ovaries, uterus, cervix, vagina and vulva were identified in the SEER*Stat 8.3.6 database, and their clinical data was retrieved. In situ malignancies were excluded. Only patients with known age and listing in the research database were included. 

Patients’ age at diagnosis, year of diagnosis, ethnicity, site, histology, stage (i.e., localized, regional and distant disease) and survival data were collected. Histologic subtypes were grouped into squamous cell carcinoma, adenocarcinoma, melanoma, sarcoma, germ-cell tumors and other subtypes/unspecified for vulvar, vaginal, cervical and uterine malignancies. Ovarian neoplasms were classified as epithelial, sex-cord-stromal germ-cell or mesenchymal tumors.

Incidence rates were adjusted for age to the 2000 United States standard population and reported per 1,000,000 women 18 years or younger. Patients with secondary gynecologic cancers were excluded from the survival analysis. The SEER data is publicly available and deidentified and therefore excluded from Ethics Board approval.

### 2.2. Statistics

Descriptive statistics were used to report demographic data. Continuous variables were compared using the Mann–Whitney-test or Wilcoxon test, as appropriate. More than two groups were compared using the Kruskal–Wallis ANOVA test. Cross-tables and Chi-square test were used to compare categorical data. The Kaplan–Meier method with log-rank test was used to analyze overall survival. Overall survival was calculated from the date of diagnosis to the date of death. Statistical analysis was performed using SPSS Version 26, IBM, Armonk, USA. A *p*-value ≤ 0.05 was considered statistically significant.

## 3. Results

Gynecologic malignancies accounted for 4.2% (1255/29,919) of all malignancies in girls ≤ 18 years and 0.3% (1255/382,510) of all gynecologic malignancies in the SEER-18 population occurred in this age group: 1.1% of all ovarian, 1.0% of all vaginal, 0.1% of all cervical, 0.1% of all vulvar and <0.1% of all uterine malignancies were diagnosed in girls ≤18 years.

The age-adjusted incidence of gynecologic malignancies and the relative frequency by organ are shown in Table 1.

**Figure 1:**
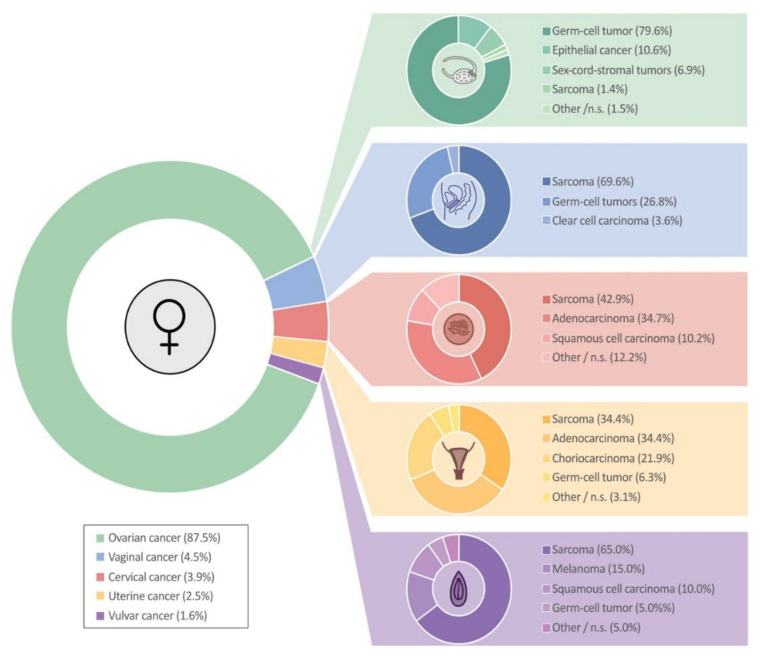
Distribution of gynecologic malignancies according to histology.

Histologic subtypes identified were germ-cell tumors (71.6%), epithelial carcinomas (12.4%), sarcomas (7.9%), sex-cord stromal tumors (6.1%) and other/unspecified subtypes (2.1%). The majority of ovarian neoplasms were germ-cell tumors, whereas sarcomas represented the most common histologic type in uterine, cervical, vaginal and vulvar malignancies. The distribution of histologic subtypes is shown in Figure 1.

Ovarian cancer accounts for 87.5% of gynecologic malignancies in children and adolescents ≤18 years, vaginal cancer 4.5%, cervical cancer 3.9%, uterine cancer 2.5% and vulvar cancer 1.6%. Abbreviations: n.s., not specified.

**Figure 2:**
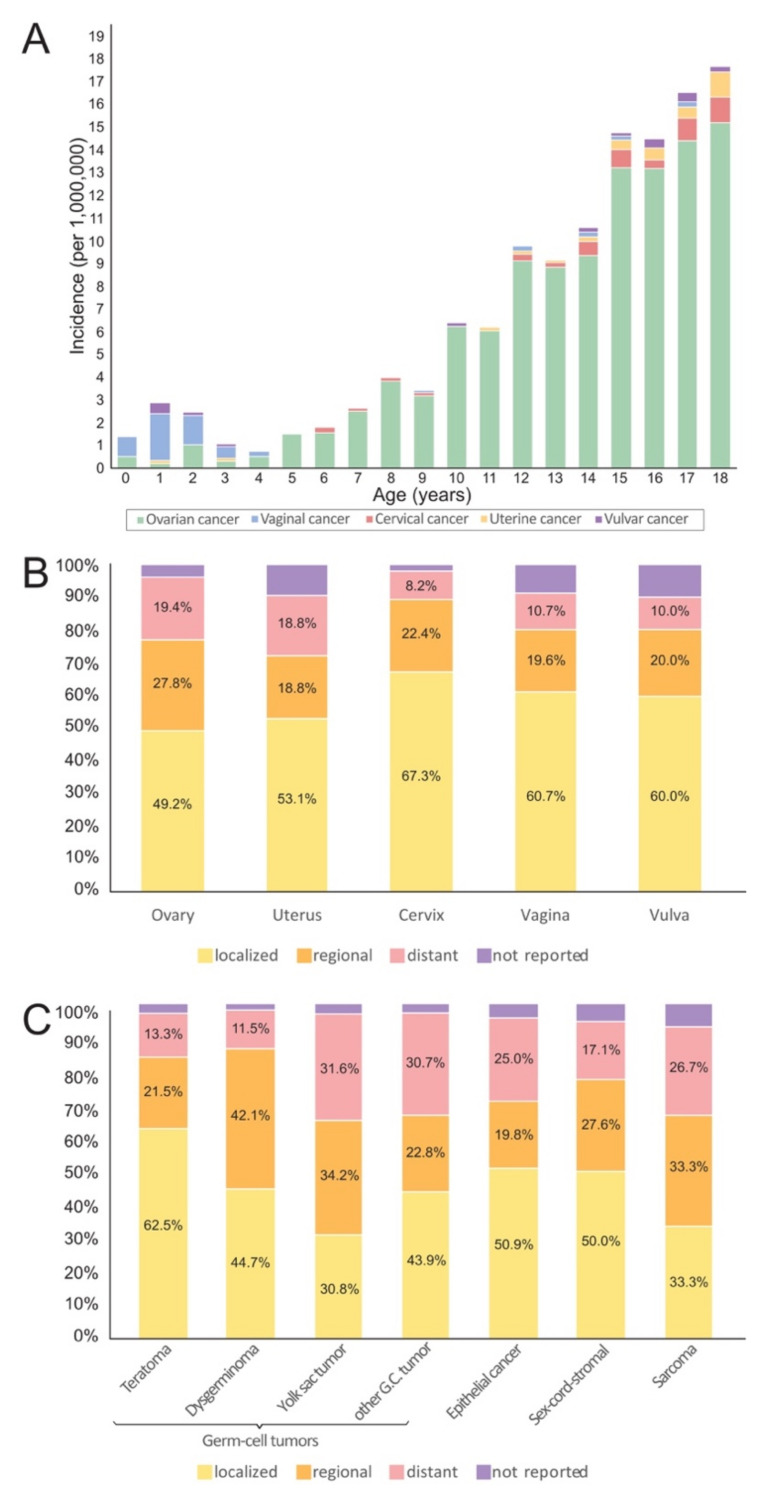
(**A**–**C**) Annual incidence of gynecologic malignancies by age from 0–18 years and distribution of disease stage at diagnosis. Abbreviations: n.a., not applicable. (**A**) The graph illustrates the annual incidence of ovarian, vaginal, cervical, uterine and vulvar cancer by age groups from 0–18 years per 1,000,000 women. (**B**) Distribution of disease stage of ovarian, vaginal, cervical, uterine and vulvar cancer in children and adolescents from 0–18 years of age. (**C**) Distribution of disease stage in ovarian malignancies by underlying histologic subtype in the same cohort. Abbreviations: G.C., germ-cell.

Age at diagnosis and ethnicity differed significantly between patients with cancers of the ovary, uterus, cervix, vagina and vulva. Age at diagnosis and the relative frequency and standardized incidence ratio of gynecologic malignancies stratified by ethnicity are shown in Table 2. Girls with vaginal cancer were significantly younger than all other organ sites (*p* < 0.001), and girls with ovarian cancer were significantly younger than those with uterine malignancies (*p* = 0.011). Age at diagnosis differed between histologic groups (*p* < 0.001). Girls with sarcomas were significantly younger than all other histologic types (*p* < 0.001). Girls with germ-cell tumors were significantly younger than girls with epithelial (*p* < 0.001), sex-cord-stromal tumors (*p* = 0.026) and other/non-specified histologic types (*p* = 0.019). The annual incidence of gynecologic cancers by age is shown in Figure 2A. Disease stage differed between gynecologic sites (*p* = 0.041) and among ovarian neoplasms (Figure 2B,C). Approximately 19% of ovarian and uterine cancers were already metastasized at the time of diagnosis. The majority of cervical, vaginal and vulvar cancers were localized.

**Figure 3:**
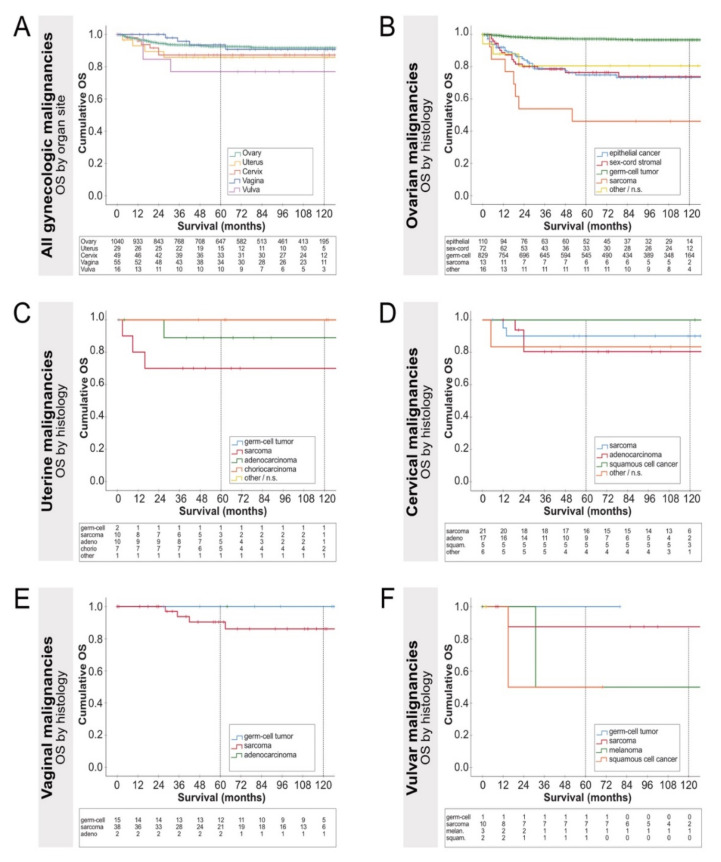
(**A**–**F**) Kaplan–Meier plots for 5- and 10-year overall survival for children and adolescents with gynecologic malignancies. (**C**) The survival curves of germ-cell tumor and choriocarcinoma are overlapping. (**E**) The survival curves of adenocarcinomas and germ-cell tumors are overlapping. Abbreviations: n.s., not specified; OS, overall survival.

The overall prognosis of gynecologic malignancies in girls ≤18 years is good. The 5-year overall survival rate for malignancies of the ovary is 92.4%, uterus 85.9%, cervix 87.2%, vagina 90.8% and vulva 76.9%. The Kaplan–Meier plot of overall survival by organ site is shown in Figure 3A and ovarian, uterine, cervical, vaginal and vulvar cancer by histologic groups in Figure 3B–F.

## 4. Discussion

In this study, we used the SEER-18 registry representing 27.8% of the US population to characterize the epidemiology, demographics and prognosis of gynecologic malignancies in children and adolescents [16]. We have shown that gynecologic malignancies account for 4.2% of all malignancies in girls ≤18 years. 1.1% of all ovarian cancers and 1.0% of all vaginal cancers in the general population are diagnosed in children and adolescents.

These tumors differ significantly from common gynecologic malignancies encountered in the general population. We have observed a significant difference in the relative frequency of these malignancies among ethnic groups (Table 2). The incidence of cervical cancer was almost three times higher in black and American Indian or Alaska native and two times higher in Hispanic than in white girls. Vaginal cancer was more common in white, black and American Indian or Alaska native than in Hispanic and Asian girls.

### 4.1. Ovarian Malignancies

While the majority of ‘’adulthood’’ ovarian malignancies are epithelial cancers [17,18], approximately 80% of all ovarian neoplasms in children and adolescents ≤18 years are germ-cell tumors (Figure 1). These cancers are observed with increasing incidence in children from 5–18 years. Although a significant proportion of yolk sac tumors, dysgerminomas and malignant teratomas are diagnosed at advanced disease stages (Figure 2B), the prognosis is excellent, with 5- and 10-year overall survival rates of 97.2% and 96.0% (Figure 3B). Ovarian sarcomas, which are often associated with a poor prognosis, represent a minority of ovarian tumors (Figure 3B). Epithelial and sex-cord-stromal tumors account for approximately 11% and 7%, respectively, and have a 10-year overall survival rate of 67.5% and 67.8%, respectively (Figure 3B). As ovarian malignancies are associated with a good prognosis, fertility-preserving treatment options must be considered for these patients. Data indicate that in appropriately selected cases, these treatments do not compromise oncological safety [19,20]. Staging and optimal patient selection are a prerequisite. Patients should therefore be referred to dedicated cancer centers to facilitate a multidisciplinary assessment and optimized delivery of care [20,21,22]. While amenorrhea is a common side-effect of certain chemotherapy regimens even in younger women, normal menstruation following primary fertility-sparing surgery and platinum-based chemotherapy has been reported in 80-99% of patients with germ-cell tumors [23,24]. In a recently published study of 105 ovarian germ-cell tumor survivors, over 90% of women <40 years of age who desired pregnancy became pregnant and gave birth [25].

### 4.2. Vaginal Malignancies

Vaginal malignancies represent the second most common gynecologic cancer in this age-group, and the majority (84%) affect children between 0 and 4 years of age (Figure 2A). Sarcomas (mainly embryonal rhabdomyosarcomas) represent the most common histologic subtype (Figure 1), are commonly diagnosed at localized disease stages and are associated with a good prognosis [26]. The 10-year overall survival rate is 86.1% (Figure 3E), which is similar to a sub-analysis of female genital tract rhabdomyomas from the Intergroup Rhabdomyosarcoma Study Group (IRSG) protocols I–IV [27] and a study combining data from five trials and registry data [11].

Germ-cell (and specifically yolk sac) tumors represent the second most common vaginal histology and have an excellent prognosis. A 10-year overall survival rate of 100% was observed in this cohort. This finding was similar to a recent single-center case series of 16 patients, where all patients, other than one who experienced a treatment-related death, had a complete response without recurrence at a median follow-up of 53 months (range, 19–223) and is similar to the outcome in malignant germ cell tumors of the ovary, where the reported mortality rate was 1.9% over a period of 20 years [14,28].

### 4.3. Cervical Malignancies

In this study, sarcomas were also found to be the most common histologic subtype of cervical neoplasms. The majority of these were embryonal rhabdomyosarcomas with a similar prognosis (Figure 3D) and treatment approach as vaginal sarcomas [26,27]. The second most common histologic subtype was adenocarcinoma, and 15 of 16 cases were cervical clear cell cancers. A strong association with intra-uterine diethylstilbestrol (DES) exposure has been shown [29], but several cases have been reported unrelated to DES, particularly in young patients [30,31,32,33].

### 4.4. Vulvar Malignancies

The majority of vulvar malignancies were sarcomas, which had a very similar prognosis to vaginal and cervical sarcomas (Figure 3E,F) and were treated in a similar fashion [27,34]. Squamous cell cancer and malignant melanoma of the vulva have a poor prognosis. Vulvar melanomas are associated with more advanced disease stages at diagnosis and worse outcomes than cutaneous melanomas [34,35,36].

### 4.5. Uterine Malignancies

Uterine malignancies are rare in childhood compared to the general population, where they represent the most common gynecologic cancer [17]. We observed a heterogenous group of sarcomas, adenocarcinomas and choriocarcinomas, the latter being more frequently found in teenagers [37]. Treatment strategies depend on the underlying histology [26].

### 4.6. Summary

This study included a large series of gynecologic malignancies in children and adolescents using the SEER-18 population and is representative of the North American population. The data are population-based and therefore not limited to single-center experiences, avoiding publication bias. The study is, however, limited by its retrospective design and the use of registry data, which preclude a central pathology review. In addition, the medical treatment approaches used in this population are not reported here. The scope of this study was to investigate the epidemiology, characteristics and real-world prognosis, and is reflected in our results.

Although gynecologic malignancies in children and adolescents are considered rare, they account for 4.2% of all malignancies in girls aged 0–18 years. Approximately 1% of all ovarian and vaginal cancers encountered in the general population affect this age group. The histologic subtypes of the reported pediatric malignancies differ from the commonly reported gynecologic cancers in the adult population. Treatment decisions should be based on a multi-disciplinary evaluation including pediatric and gynecologic oncologists. With regards to ovarian cancers, germ cell tumors represent the most common ovarian neoplasm. In vulvo-vaginal, uterine and cervical malignancies, sarcomas represent the most common subtype compared to squamous and adenocarcinomas in the adult populations. Overall survival in this study was found to be excellent; therefore, counseling for fertility preserving therapies is encouraged.

## Figures and Tables

**Table 1:** Age-adjusted incidence and relative frequency of gynecologic malignancies among children and adolescents 0–18 years of age.

Type of Malignancy	Incidence per 1,000,000 Women (95% CI)	Relative Frequency %
All gynecologic malignancies	6.719 (6.352–7.101)	100%
Ovarian malignancies	5.882 (5.540–6.241)	87.5%
Uterine malignancies	0.170 (0.116–0.240)	2.5%
Cervical malignancies	0.262 (0.193–0.346)	3.9%
Vaginal malignancies	0.298 (0.225–0.388)	4.5%
Vulvar malignancies	0.106 (0.065–0.164)	1.6%

Incidence adjusted to the 2000 United States standard population. Relative frequency of cancer among all gynecologic malignancies in children and adolescents 0–18 years of age.

**Table 2:** Demographic characteristics by organ.

Parameter	Ovary (*n* = 1098)	Uterus (*n* = 32)	Cervix (*n* = 49)	Vagina (*n* = 56)	Vulva (*n* = 20)	*p*
**Age (years)**						
Mean age ± SD	13.7 ± 3.7	15.3 ± 4.0	15.0 ± 3.2	3.4 ± 4.8	11.5± 6.9	<0.001
Median age (range)	15.0 (0–18)	16.5 (1–18)	16.0 (6–18)	1.5 (0–17)	15.5 (1–18)	
**Ethnicity (%)**						
White	41.0%	43.8%	30.6%	58.9%	45.0%	0.029
Black	13.9%	12.5%	26.5%	17.9%	5.0%	
Hispanic	33.8%	40.6%	36.7%	16.1%	45.0%	
Asian or Pacific Islander	9.5%	3.1%	0.0%	5.4%	5.0%	
American Indian or Alaska Native	0.6%	0.0%	2.0%	1.8%	0.0%	
Unknown	1.2%	0.0%	4.1%	0.0%	0.0%	
**Ethnicity (Incidence per 1,000,000**)						
White	4.966	0.151	0.164	0.383	0.098	
Black	5.830	0.153	0.488	0.400	0.040	
Hispanic	7.148	0.253	0.346	0.153	0.167	n.a.
Asian or Pacific Islander	6.515	0.061	0	0.186	0.062	
American Indian or Alaska Native	3.462	0	0.499	0.498	0	

Incidence adjusted to the 2000 United States standard population. Abbreviations: SD, standard deviation.

## Data Availability

The SEER data is publicly available [16].
